# The Role of *Trichoderma harzianum* Elicitor *Hyd1* in Inducing the Maize Endophytic Microbial Community and *Bacillus* Strains Against Maize Root Rot

**DOI:** 10.3390/jof12060395

**Published:** 2026-05-30

**Authors:** Gaoyue Si, Xifen Zhang, Cheng Zhang, Yaqian Li, Xinhua Wang, Ning Guo, Jie Chen

**Affiliations:** 1 School of Agriculture and Biology, Shanghai Jiao Tong University, Shanghai 200240, China; 2 The State Key Laboratory of Microbial Metabolism, Shanghai Jiao Tong University, Shanghai 200240, China; 3 Plant Protection Institute, Hebei Academy of Agriculture and Forestry Sciences, National Collection of Plant-Associated Microbes (Hebei), IPM Innovation Center of Hebei Province, International Science and Technology Joint Research Center on IPM of Hebei Province, Baoding 071000, China; 4 Inner Mongolia Research Institute, Shanghai Jiao Tong University, Hohhot 010010, China

**Keywords:** hydrophobin, *Trichoderma*, root rot, induced resistance

## Abstract

Fusarium root rot (caused by *Fusarium verticillioides*) is a destructive soilborne disease in maize, significantly reducing crop yields. The root symbiotic fungi *Trichoderma* species have been confirmed as effective biocontrol microbes for Fusarium root rot; however, the mechanistic role of *Trichoderma*-induced endophytes in suppressing Fusarium root rot in maize remains unclear. This study found that *Trichoderma harzianum* T30 significantly reduced the abundance of pathogens by 48.9% and increased the abundance of potentially antagonistic *Bacillus* strains (33%) in the root endophytic bacterial community. In addition, the *hyd1* gene in *T. harzianum* T30 induced a 7.5-fold upregulation of *ZmOPR7* in maize roots compared to the Δ*hyd1* mutant treatment, a gene related to the jasmonic acid (JA) pathway. Further, several endophytic *Bacillus* strains were specifically induced by a *hyd1*-overexpressing strain, including *B. amyloliquefaciens* MX66, *B. velezensis* C9, and *B. velezensis* GAGAN3. Three endophytes significantly (*p* < 0.05) reduced Fusarium root rot incidence in maize by 46.6–55.0% and upregulated the expression of jasmonic acid/ethylene (JA/ET) pathway-related genes (*ZmOPR7*, *ZmOPR8* and *ZmEIL1*) by 5.4-, 1.5-, and 4.6-fold, respectively, compared to untreated controls. Meanwhile, the *Bacillus* strain also improved maize plant growth. This study examined how overexpression of the *T. harzianum* elicitor gene *hyd1* (in the OE-*hyd1* strain) affects the colonization dynamics of beneficial endophytic bacteria in maize roots. Additionally, it further suggested the contribution of selected endophytic *Bacillus* strains in suppressing Fusarium root rot in maize.

## 1. Introduction

Maize (*Zea mays* L.), one of the most economically significant cereals, is widely grown, with an annual production of 850 million metric tons, providing food to billions of people worldwide [1,2,3]. Maize root rot, caused by *Fusarium* spp., with the main fungal phytopathogen being *Fusarium graminearum* and *Fusarium verticillioides*, leads to the loss of yield and quality, which seriously threatens maize agriculture on a global scale [4]. *Fusarium verticillioides* (*Fv*) could infect maize plants at the seed and seedling stage [5,6].

Currently, conventional chemical control, mainly using chlorothalonil, mancozeb, and carbendazim, is commonly used to manage root rot [7,8,9]. However, the severe drawbacks of prolonged chemical fungicide use leave people with ecological issues. In this sense, biological control agents (BCAs) have been widely viewed as eco-friendly, cost-effective, and bio-fungicides effective against a vast array of agriculturally destructive soilborne phytopathogens [10,11,12,13,14]. *Trichoderma* has also been reported to be effective as a biocontrol fungus against maize soilborne diseases, including root rot and stalk rot caused by *Fusarium* species; it has also been found that *Trichoderma* can induce changes in the endophytic bacteria in maize plants during the control of soilborne diseases [15,16,17]. Among biocontrol agents, *Bacillus* species have demonstrated promising efficacy in suppressing root rot pathogens and enhancing crop yield stability, while simultaneously reducing reliance on synthetic pesticides in agricultural management [18,19,20,21,22].

For instance, *Bacillus subtilis* RA15 and *Bacillus tequilensis* FC6 have been reported to have antagonistic activity against *F. verticillioides* and to enhance plant growth [23]. Both bacterial strains show significant growth inhibition of *F. verticillioides* in vitro. Furthermore, their lipopeptide extracts, even at the lower concentration tested (10 mg/L), especially those from *B. tequilensis* FC6, can significantly protect against the disease comparably to the chemical control (55% and 51% for strain FC6 and the control, respectively) [23]. A previous study suggests that endophytic *Bacillus* strains inhibit sheath blight [22]. Similarly, *Bacillus* species are used as biological control agents to combat plant diseases and are of particular importance in controlling soilborne diseases, including root rot caused by *F. verticillioides* [20,22,24,25]. The microbial community composition and interactions were regulated by biocontrol bacteria strains (*Bacillus subtilis* and *Bacillus amyloliquefaciens*) against diseases, and improved plant growth, and microbial diversity facilitates the suppression of Fusarium diseases [2,8,11,15,26].

Previous studies found that overexpression of the elicitor gene *hyd1* in *T. harzianum* enhanced root colonization and induced systemic resistance against *Curvularia* leaf spot, and this resistance was positively correlated with the activation of brassinosteroid (BR) and jasmonate/ethylene signaling pathways [27]. Moreover, *hyd1* can function as an elicitor in *Trichoderma* and is recognized by the plant’s pattern recognition receptors (PRRs), triggering maize’s basal defense response. The contribution of *Trichoderma*-induced endophytic bacteria to the control of Fusarium root rot and the role of the elicitor released from *Trichoderma* in reshaping the endophytic community in root tissues for inhibiting root rot pathogen infection is not fully understood. Understanding the association between *Trichoderma* elicitor, microbial community diversity, and induced beneficial microbes will facilitate microbial control applications in agriculture. In this study, we first investigated the interrelationship between the overexpression of the *hyd1* gene in the OE-*hyd1* strain of *T. harzianum* T30 and its ability to colonize maize seedlings, and the changes in the root endophytic microbial diversity related to the control of Fusarium root rot in maize, and further screened some endophytic bacteria that can inhibit Fusarium infection in the root. This study provides support for the biological control of Fusarium root rot. By investigating the synergistic role of the *T. harzianum* elicitor *hyd1* and the maize root microbial community associated with endophytic *Bacillus* strains, we aim to explore alternative biological control strategies in the future.

## 2. Materials and Methods

### 2.1. Strains and Plant Materials

*T. harzianum* strain T30 (CGMCC No. 22479), *hyd1*-knockout (KO16) and overexpression (OE) strains [27], were cultured on potato dextrose agar (PDA) in 90 mm diameter Petri dishes at 28 °C for 7 days (Supporting Information: Figure S1). The Δ*hyd1* knockout mutants were generated using the *Agrobacterium tumefaciens*-mediated transformation (ATMT) method. A modified pCAMBIA1300qh vector carrying the Hygromycin B resistance gene was used, with approximately 1 kb upstream and downstream sequences of *hyd1* serving as homologous recombination arms [27]. Total DNA was extracted from putative transformants using the CTAB method, and PCR verification confirmed two independent knockout mutants, designated as KO16. The OE-*hyd1* strain was constructed based on the wild-type T30 genome. Using fusion PCR, the trpC promoter, *hyd1* CDS region, and trpC terminator were connected to form an overexpression cassette. The correctly fused fragment was digested with *XbaI* and *BamHI* and ligated into a modified pCAMBIA1300th vector. Overexpression transformants were obtained via *Agrobacterium tumefaciens*-mediated transformation (ATMT). Genomic DNA was extracted for validation. The expression of OE-*hyd1* was higher than that of the wild-type T30, while the reference gene Actin showed consistent expression across samples, confirming the successful construction of the overexpression strain [27]. *Trichoderma* strains’ spores were harvested by washing the culture with sterile water and filtering it through four layers of sterile gauze, and the spores were counted using a hemocytometer to adjust the concentration to 10^6^ CFU/mL.

Maize seeds (cultivar Zhengdan 958) were treated by soaking in hot water at 40 °C for 3 h, surface-sterilized with 10% sodium hypochlorite for 11 min, rinsed 2–3 times with sterile water, and further disinfected with 70% ethanol for 65 s followed by six washes with sterile water. The spore suspension (10^6^ CFU/mL) was mixed with a 1% sodium carboxymethyl cellulose (CMC) solution, and the seeds were coated with this mixture. The pathogen *F. verticillioides* (*Fusarium verticillioides* 7600) was preserved at Shanghai Jiao Tong University. The pathogen *F. verticillioides* was first activated on potato dextrose agar (PDA) at 28 °C for 7 d. Mycelial plugs (2 mm diameter) from the colony edge were aseptically transferred to autoclaved wheat seeds (moisture content adjusted to 30%). The inoculum was incubated at 28 °C for 5 d until full colonization. Fusarium-colonized wheat grains (30 seeds per pot) were planted into the zone of maize seeds in pots (15 cm in height and 12 cm in diameter).

The pot experiments were designed with two groups. Group 1 had four treatments without pathogen inoculation, including control seedlings without any inoculum (CK), seedlings inoculated with *Trichoderma* strain T30 (wild type), seedlings inoculated with the *hyd1*-knockout mutant of T30 (KO16), and seedlings inoculated with *hyd1*-overexpressing T30 (OE-*hyd1*). In Group 2, the seedlings received identical microbial treatments to Group 1 (CK, T30, KO-*hyd1*, OE-*hyd1*) but were additionally challenged with *F. verticillioides*. There were four treatments in a pot experiment under the pathogen inoculation system: (i) seedlings were infected with *F. verticillioides* only (CK + *F. v*); (ii) seedlings were preinoculated with T30 and then infected with *F. verticillioides* (T30 + *F. v*); (iii) seedlings were preinoculated with KO16 and then infected with *F. verticillioides* (KO16 + *F. v*); and (iv) seedlings were preinoculated with OE-*hyd1* and then infected with *F. verticillioides* (OE-*hyd1* + *F. v*). Six replicates per treatment were established, and each replicate consisted of four seedlings.

After 4 weeks, root rot disease severity was scored according to the following grading scale. Grade 0: immune, no symptoms; Grade 1: mild infection with a rot area ≤ 10% of root tissue; Grade 2: moderate infection with an affected area of 10–30%, with no rotten branches or fibrous roots; Grade 3: advanced infection with a rot area of 30–50%, with rot of branches and fibrous roots; Grade 4: severe infection with a rot area of 50–70%, with rot of branches and fibrous roots; Grade 5: complete necrosis with a rot area >70% or fully rotted roots, and the plant being dead with rot of branches and fibrous roots. The disease index was calculated using the following formula: Disease index = [∑(level *n* × number of diseased plants at level *n*)/(total checked plants × 6)] × 100. Here, *n* represents the grading level. Control effect = [(control average disease index-treatment average disease index)/control average disease index]  × 100%.

### 2.2. 16S rRNA Sequencing and Microbial Community Analysis of Maize Root

To determine whether *hyd1* mutants of *Trichoderma* strain T30 or Fusarium infection influence the maize root bacterial community, inoculated root samples were collected for *16S* rRNA gene sequencing of the endophytic bacteria. There were eight treatments (CK, maize without inoculation; T30, with *T. harzianum* inoculation; KO16, with KO-*hyd1* inoculation; OE, with *T. harzianum* OE-*hyd1* inoculation. CK-FV, with *F. verticillioides* inoculation; T30-FV, co-inoculation with *T. harzianum* and *F. verticillioides*; KO16-FV, co-inoculation with KO-*hyd1* and *F. verticillioides*; OE-FV, co-inoculation with *T*. *harzianum* OE-*hyd1* and *F. verticillioides*) in the experiment. Specifically, maize root samples with three replicates per treatment were harvested and sterilized in 75% (*v*/*v*) ethanol for 1 min and 2.5% sodium hypochlorite for 10 min, before being rinsed with sterile water three times for subsequent high-throughput sequencing analysis.

Freeze-dried maize root samples were subjected to DNA extraction. The V5–V7 hypervariable region of the bacterial *16S* rRNA gene was amplified via PCR using the barcoded primers 799F (5′-AACMGGATTAGATACCCKG-3′)–1193R (5′-ACGTCATCCCCACCTTCC-3′). The PCR mix (25 μL) contained 5 μL of 5× Reaction Buffer, 1 μL of each primer (10 μM), 5 μL of 5× High GC Buffer, 2 μL of dNTPs (10 mM), 0.25 μL of Q5 high-fidelity DNA polymerase, 2 μL of template DNA, and 8.75 μL of ddH_2_O. The PCR protocol consisted of initial denaturation at 98 °C for 5 min; 25 cycles of denaturation (98 °C, 30 s), annealing (53 °C, 30 s), and extension (72 °C, 45 s); and a final extension at 72 °C for 5 min. The extracted DNA products were verified using a NanoDrop NC2000 spectrophotometer (Thermo Fisher Scientific, Waltham, MA, USA) and 2% agarose gel electrophoresis, respectively. *16S* rDNA amplicon libraries were sequenced on the Illumina NovaSeq-PE250 platform (Personalbio Inc., Shanghai, China).

The sequencing data were processed using QIIME 2 (Quantitative Insights into Microbial Ecology 2 (version 2024.5)) for microbial bioinformatic analysis (Supporting Information: Table S2). The raw sequence data were demultiplexed using the demux plugin, followed by primer cutting with the Cutadapt plugin. The sequences were then quality-filtered, denoised, merged, and chimera-removed using the DADA2 plugin. Non-singleton amplicon sequence variants (ASVs) were aligned with mafft and used to construct a phylogeny with fasttree2. Alpha diversity metrics (Chao1, Shannon, Simpson) and beta diversity metrics (Bray–Curtis dissimilarity) were determined using the diversity plugin with sample rarefaction. Taxonomy was distributed to ASVs using the classify-sklearn naive Bayes taxonomy classifier in the feature-classifier plugin against the SILVA Release 138 Database. ASV-level alpha diversity indices, including the Chao1 richness estimator, Shannon diversity index, and Simpson index, were calculated using the ASV table in QIIME2, and visualized as box plots. Beta diversity analysis was performed to investigate the structural variation in microbial communities across samples using the Bray–Curtis metric and visualized via principal coordinate analysis (PCoA). An upset diagram was generated to visualize the shared and unique ASVs among samples. PERMANOVA (permutational multivariate analysis of variance, with the ADONIS matrix and 999 permutations) was used to analyze the significance of microbiota structure differentiation among groups via QIIME2 in R (version 4.3.3). The differentially abundant taxa across groups were detected by LEfSe (linear discriminant analysis effect size) analysis with the default parameters (LDA score ≥ 2.0, with Kruskal–Wallis test, *p* < 0.05). PICRUSt2 (Phylogenetic investigation of communities by reconstruction of unobserved states, version 2.2.2-b, NTSI < 2, with Z score row standardization) was used to predict microbial functions using the MetaCyc (https://metacyc.org/, accessed on 12 June 2025) databases.

### 2.3. Isolation and Identification of Antagonistic Bacteria

Bacteria were isolated concurrently with the collection of maize root samples for microbiota sequencing. Selected root samples (8 mm diameter) were rinsed with tap water to remove the soil particles, and then immersed in a 70% (*v*/*v*) ethanol solution for 65 s; these samples were sterilized with 2.5% sodium hypochlorite for 5 min and finally rinsed again with distilled water six times. To assess the efficiency of the surface disinfection, 0.2 mL of the final rinse water was used to inoculate LB (Luria–Bertani) agar medium [28].

The surface-disinfected roots were transferred and ground in a sterile mortar, and one-to-ten serial dilutions were made by mixing ground fresh root homogenate with 10 mL of sterile distilled water. After mixing, a 10^−2^ diluted suspension was obtained, and subsequently, it was serially diluted to 10^−3^, 10^−4^, 10^−5^, and 10^−6^ suspensions. Next, 0.2 mL of the root suspension of each concentration was plated on LB agar, with three replicates per gradient, and incubated upside down at 28 °C for 3–7 days. A single colony was selected and streaked on new LB plates for purification. All the purified, isolated strains were preserved in sterilized glycerol and stored in an ultra-low-temperature freezer at −80 °C. To screen for antagonistic bacterial strains, the tested bacterial strains were cultivated in 200 mL of LB broth in shake flasks at 37 °C and 180 rpm overnight. Then, 10 mL of bacterial supernatant (the fermentation broth was filtered through a 0.22 μm sterile filter) was added to the potato dextrose agar medium (20 mL). A 4 mm pathogen agar plug of *F. verticillioides* and *Exserohilum turcicum* was inoculated in the center of the composite medium plate. Plates inoculated with the pathogen but not inoculated with the bacterial test strains were used as a control check (CK) treatment, and each plate of each treatment was replicated three times. The effectiveness of each tested bacterial strain was evaluated after 7 days of incubation. The inhibition rate = [(control colony diameter-treatment colony diameter)/control colony diameter] × 100%.  Bacterial strains were identified using PCR amplification of the primers 27F (5′-AGRGTTYGATYMTG¬GCTCAG-3′) and 1492R (5′-YGRTACCTTGTTAC¬GACTT-3′). The amplified products were confirmed by 1% gel electrophoresis. The amplified samples and appropriate sequencing fragments were sent for sequencing. Bacterial isolates were further classified at the species level by BLAST (https://blast.ncbi.nlm.nih.gov/Blast.cgi, accessed on 17 June 2025) search using GenBank NCBI (National Center for Biotechnology Information).

### 2.4. Control Efficiency Assay in the Greenhouse

The pot experiment was performed in the greenhouse. All bacterial strains were cultured using the shake flask fermentation method [29] in LB medium at 37 °C. Each strain was adjusted to an OD_600_ value of 0.05. The surface-sterilized germinated seeds were immersed in different bacterial suspensions for 2 h, and the seed immersed in sterile water was used as a control group. Treated seeds were planted and infected through *F. verticillioides* grain inoculation. Two control groups were established—CK (the uninoculated group) and the group inoculated with the pathogen—and three different treatment groups were set up, with each inoculated with one of the three bacterial strains and the pathogen. There were five treatments in the experiment: (i) non-inoculated treatment (CK); (ii) seedlings infected with *F. verticillioides* only (*F. v*); (iii) seedlings preinoculated with *Bacillus amyloliquefaciens* MX66 and then infected with *F. verticillioides* (*B. amyloliquefaciens* MX66 + *F. v*); (iv) seedlings preinoculated with *Bacillus velezensis* C9 and then infected with *F. verticillioides* (*B. velezensis* C9 + *F. v*); (v) seedlings preinoculated with *B. velezensis* GAGAN3 and then infected with *F. verticillioides* (*B. velezensis* GAGAN3 + *F. v*). There were six replicates per treatment, and each replicate included four seedlings. Subsequently, the disease index was recorded, and the maize plant height, root length, and root weight were measured for different treatments.

### 2.5. Detection of Host Defense Gene Expression

Maize seedling roots, both infected and non-infected ones, were sampled with three replicates. Then, 0.2 g of plant tissue was placed in 2 mL RNase-free Eppendorf bottles and ground into powder in a mortar. Based on the test method of the RNA extraction kit, 500 μL of buffer PRL was added, and the mixture was vortexed for 30–60 s. The lysate was placed in a water bath for 5 min and then centrifuged at 12,000 rpm for 10 min. The supernatant was transferred into new 1.5 mL RNase-free tubes. An equal volume of absolute ethanol (50% of the total volume with respect to the supernatant) was added to the tubes, and the solution was mixed by pipetting. The mixed solution was transferred into FastPure gDNA-Filter Column II tubes and centrifuged at 12,000 rpm for 2 min. The filtrate was discarded. The FastPure gDNA-Filter Column II tubes were placed in new 2 mL collection tubes, 500 μL of buffer PRLPlus was added, and the tubes were centrifuged at 12,000 r/min for 30 s. Then, the filtrate was collected. Another equal volume of absolute ethanol (50% of the total volume with respect to the supernatant) was added to the collected filtrate, and the solution was immediately mixed by pipetting.

The mixed solution was transferred into FastPure RNA Column IV tubes and centrifuged at 12,000 r/min for 2 min, and the filtrate was discarded. A 700 μL volume of buffer PRW1 was added to the column, and then it was centrifuged at 12,000 r/min for 30 s, and the filtrate was discarded. A 500 μL volume of buffer PRW2 was added to the column, and it was centrifuged at 12,000 rpm for 30 s. The filtrate was discarded, and this procedure was repeated. The column was centrifuged at 12,000 rpm for 2 s to remove any remaining liquid. The FastPure RNA Column IV tubes were transferred into new 1.5 mL RNase-free tubes, and 50 μL of RNase-free ddH_2_O was added to the center of the absorption column with free space. The tube was placed at room temperature for 2 min, and then centrifuged for 2 min, and centrifuged at 12,000 r/min for 1 min.

Reverse transcription assay: The mixture was prepared in RNase-free tubes; 4 μL of 4× gDNA wiper Mix, 1 pg–1 μg of RNA template, and RNase-free ddH_2_O were added to a final volume of 16 μL. The solution was mixed by pipetting and incubated at 42 °C for 2 min. The reverse transcription system was configured by adding 4 μL of 5xHiScript III Qrt SuperMix to the tubes, and the solution was immediately mixed by pipetting. The solution was incubated in a water bath at 37 °C for 15 min and then at 85 °C for 5 s, and placed in the qPCR system. The reaction system was configured according to the qRT-PCR test kit instructions: 10 μL of 2× ChamQ Universal SYBR qPCR Master Mix, 0.4 μL of Primer1, 0.4 μL of Primer2, 1 μL of DNA/cDNA template, and 8.2 μL of ddH_2_O. The primers of the experiment are listed in Table S1 [27]. The qRT-PCR amplification system was as follows: Pre-denaturation at 95 °C for 30 s. Amplification cycles: denaturation at 95 °C for 10 s, annealing for 15 s, and extension at 60 °C for 30 s, with 40 cycles. Melting curve analysis: temperature rising to 95 °C for 15 s, decreasing to 60 °C for 60 s, and then increasing to 95 °C for 15 s, and the melting curve was drawn. The data were collected with three replicates, and the 2^−ΔΔCt^ method was used to calculate the relative expression of genes.

### 2.6. Data Analysis

The Kruskal–Wallis test was used to assess significant differences in alpha diversity among groups. PERMANOVA was used to examine the significant differences in microbial communities (beta diversity) across the sample groups. Genus-level biomarker features were identified in each group using the linear discriminant analysis (LDA) of the effect size (LEfSe) method according to LDA scores (log10) ≥ 2.0 and *p* < 0.05. Root bacterial marker-gene (*16S* rRNA) amplicon sequences were used for functional prediction with PICRUSt2 using the MetaCyc databases. Statistical and graphical representations of the test data in the pot experiment were generated using SPSS 16.0 and GraphPad Prism 9. One-way analysis of variance (ANOVA), Tukey’s test, and Duncan’s new multiple-range test were used for significance analysis of the data, and the results are expressed with different lowercase letters, where different letters indicate significant differences at a certain significance level.

## 3. Results

### 3.1. The Hyd1 Gene from T. harzianum Strain T30 Promotes Maize Growth and Confers Resistance to Fusarium Root Rot Under Greenhouse Conditions

**Figure 1:**
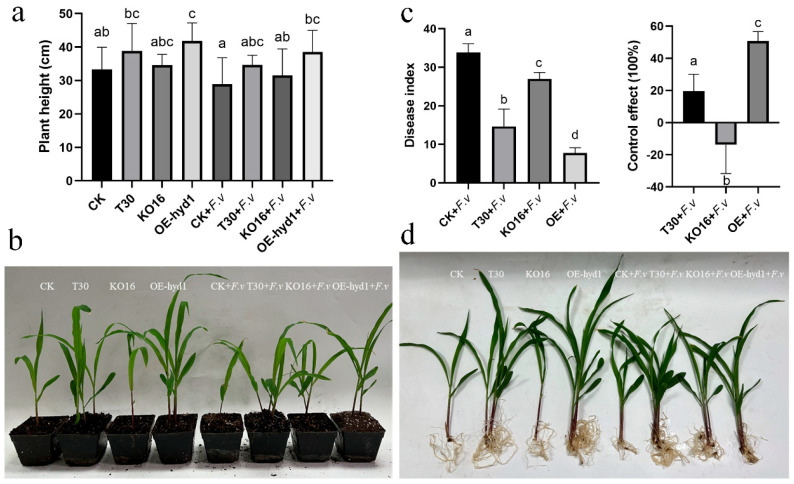
The effects of different treatments on plant growth and disease inhibition. (**a**) Plant height of maize in different treatments (CK, maize without inoculation; T30, the wild-type (WT) strain of *T. harzianum*, which naturally expresses *hyd1* endogenously; KO16, the *hyd1*-knockout strain; OE-*hyd1*, a strain that overexpresses *hyd1* in *T. harzianum*, which was generated in the T30 wild-type background. CK + *F.v*, with *F. verticillioides* inoculation; T30 + *F.v*, co-inoculation with *T. harzianum* and *F. verticillioides*; KO16 + *F.v*, co-inoculation with KO-16 and *F. verticillioides*; OE-hyd1 + *F.v*, co-inoculation with *T*. *harzianum* OE-*hyd1* and *F. verticillioides*). (**b**) The control effect and disease index of the treatments. (**c**) Phenotypes of maize in different treatments. (**d**) Disease severity of maize root rot in different treatments. Values are means ± SDs (*n* = 3). Different letters indicate a significant difference according to Tukey’s test *(p* < 0.05).

We first evaluated the effects of the *Trichoderma* wild-type (T30), Δ*hyd1* mutant (KO16), and OE-*hyd1* strains on maize growth at the seedling stage (3–5 leaves) in a pot experiment. The OE-*hyd1* strain significantly promoted plant growth, resulting in a seedling height that was 10% and 22.4% greater than that in the T30 and Δ*hyd1* treatments, respectively, regardless of Fusarium challenge (Figure 1a). We next assessed the control efficacy of these strains against Fusarium root rot. The OE-*hyd1* strain demonstrated superior biocontrol activity, with a control efficacy that was 24.3% and 46.8% higher than that of the wild-type T30 and the Δ*hyd1* mutant, respectively (Figure 1b).

**Figure 2:**
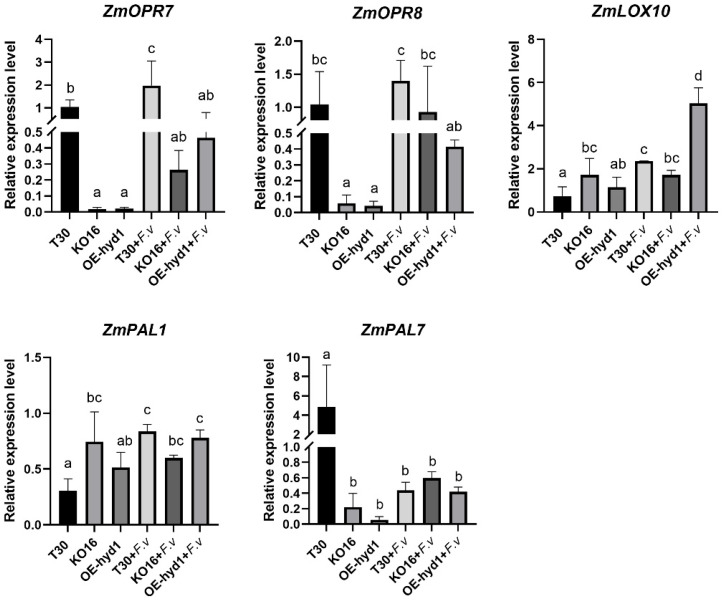
Relative expression of *ZmOPR7*, *ZmOPR8*, *ZmLOX10, ZmPAL1*, and *ZmPAL7* genes in maize roots with different inoculation treatments. Data represent the means ± SDs (*n* = 3). Error bars represent SDs. Different letters indicate significant differences (*p* < 0.05) based on Tukey’s least significant difference test.

To investigate the underlying defense mechanisms, we analyzed the expression of key genes in maize defense signaling pathways following *F. verticillioides* infection (Figure 2). The expression levels of jasmonic acid (JA) pathway-related genes (*ZmLOX10*, *ZmOPR7*, and *ZmOPR8*) were significantly higher in roots treated with the wild-type T30 strain compared to the Δ*hyd1* mutant, indicating that the *hyd1* gene positively regulates root defense. This induction was even more pronounced in the OE-*hyd1* treatment, where the expression of *ZmLOX10* and *ZmOPR7* was enhanced by 2.9-fold and 1.7-fold, respectively, relative to the Δ*hyd1* mutant treatment. In contrast, the expression of *ZmPAL7*, a gene involved in the salicylic acid pathway, was upregulated in the Δ*hyd1* mutant treatment compared to both T30 and OE-*hyd1* under pathogen challenge. Collectively, these results demonstrated that the elicitor protein *Hyd1* encoded by the *hyd1* gene in T30 suppresses Fusarium root rot primarily by activating the JA-dependent defense pathway in maize roots.

### 3.2. The Hyd1-Overexpressing T. harzianum Strain Alters the Composition and Diversity of the Root Endophytic Bacterial Community

**Figure 3:**
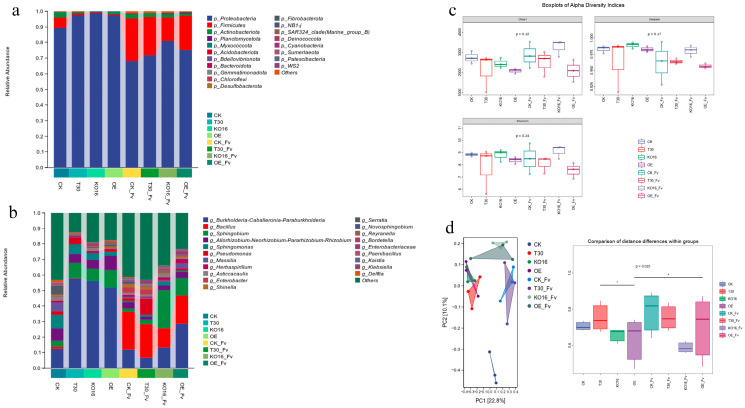
Effects of *hyd1*-mutant strains of T30 and *F. verticillioides* inoculation on the composition and diversity of the endophytic bacteria of maize roots. Taxonomic structure of the root bacterial community at the phylum (**a**) and genus (**b**) levels under different *hyd1*-mutant treatments and infection conditions. (**c**) Alpha diversity metrics of maize root endophytic bacteria based on the Chao1 index, Simpson index, and Shannon index. (**d**) Principal coordinate analysis plot of endophytic bacterial community composition and comparison analysis of distance differences within groups based on the Bray–Curtis distance for different *hyd1* treatments and infection conditions. (CK, maize without inoculation; T30, with *T. harzianum* inoculation; KO16, with KO-*hyd1* inoculation; OE, with *T. harzianum* OE-*hyd1* inoculation. CK_Fv, with *F. verticillioides* inoculation; T30_Fv, co-inoculation with *T. harzianum* and *F. verticillioides*; KO16_Fv, co-inoculation with KO-*hyd1* and *F. verticillioides*; OE_Fv, co-inoculation with *T*. *harzianum* OE-*hyd1* and *F. verticillioides*). Values are means ± SDs (*n* = 3). Asterisks indicate a significant difference according to Tukey’s test (*p* < 0.05). Differences in bacterial beta diversity among different *hyd1* mutants and infection conditions according to analysis of permutational multivariate analysis of variance. A box plot displays the first and third quartiles, with the horizontal bar at the median and whiskers showing the most extreme data points, which are no more than 1.5 times the interquartile range from the box.

Analysis of the root endophytic bacterial communities revealed that *Proteobacteria*, *Firmicutes*, and *Actinobacteriota* were the dominant phyla across all treatments inoculated with the wild-type T30 or the Δ*hyd1* mutant strain, regardless of *F. verticillioides* co-inoculation (Figure 3a). At the genus level, the community composition was notably shifted in treatments involving the *T. harzianum* OE-*hyd1* strain. Specifically, *Pseudomonas*, *Sphingomonas*, *Flavobacterium*, and *Streptomyces* were more abundant in these treatments, both with and without *F. verticillioides* (Figure 3b). This reshaping effect was particularly evident under pathogen challenge. When plants were co-inoculated with the OE-*hyd1* strain and *F. verticillioides*, the relative abundance of the beneficial genera *Bacillus* and *Pseudomonas* increased significantly at the seedling stage. Notably, the relative abundance of endophytic *Bacillus* increased by 33% in this co-inoculation treatment. While no significant differences in Shannon’s diversity index were observed among the treatments (Figure 3c), the bacterial community structure was significantly altered. Permutational multivariate analysis of variance (PERMANOVA) based on Bray–Curtis distances confirmed that the treatment type was a significant factor driving microbiota assembly. Specifically, colonization by the *T. harzianum* OE-*hyd1* strain distinctively shaped the root bacterial community structure (Figure 3d).

### 3.3. Trichoderma harzianum OE-Hyd1 Shapes the Maize Root Endophytic Bacterial Community

**Figure 4:**
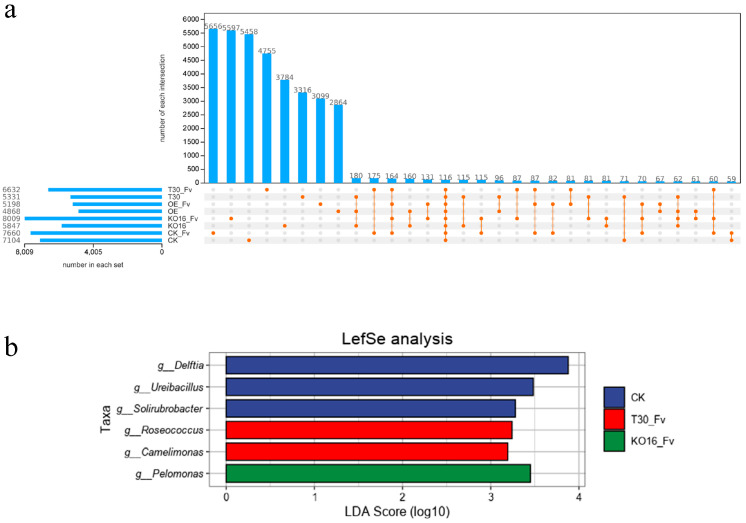
The differences in the community of endophytic bacteria in maize roots at the genus level under different *T. harzianum hyd1* treatments and infection conditions. (**a**) Upset diagram analysis depicting the number of bacterial ASVs in maize roots. The bar chart on the left depicts the bacterial ASVs from the different treatments; the line plot with points at the bottom right presents the intersection of bacterial ASVs among different treatments, the solid orange points in the graph represent that some bacterial ASVs were shared from different treatments, and the gray points show that the bacterial ASVs of different treatments have no intersection. The column chart above presents the relevant common bacterial ASVs corresponding to the intersection. CK, maize without inoculation; T30, with *T. harzianum* inoculation; KO16, with KO-*hyd1* inoculation; OE, with *T. harzianum* OE-*hyd1* inoculation. CK_Fv, with *F. verticillioides* inoculation; T30_Fv, co-inoculation with *T. harzianum* and *F. verticillioides*; KO16_Fv, co-inoculation with KO-*hyd1* and *F. verticillioides*; OE_Fv, co-inoculation with *T*. *harzianum* OE-*hyd1* and *F. verticillioides*. (**b**) Taxonomic differences among root endophytic bacteria were determined via the linear discriminant analysis (LDA) effect size (LEfSe) method, and the LDA threshold was 2.0. The X-axis represents the LDA score, and the greater the LDA score, the greater the contribution of the taxon to differences between groups. The Y-axis lists different taxa at the genus level, including unclassified ones.

To assess the impact of the *T. harzianum* elicitor gene *hyd1* on the maize root bacterial community, we inoculated pot-grown maize with the wild-type strain T30, its *hyd1*-overexpressing mutant (OE-*hyd1*), and its Δ*hyd1* mutant (KO16). Core microbial community analysis revealed that 180 core bacterial ASVs coexisted in all three treatment groups (Figure 4a). The total number of ASVs identified in each treatment was 5531 (T30), 4868 (OE-*hyd1*), and 5847 (KO16). Furthermore, we assessed the bacterial communities when maize was co-inoculated with *F. verticillioides* and different strains of *T. harzianum*. The number of bacterial ASVs identified was 6632 for the wild-type T30 co-inoculation, 5198 for the OE-*hyd1* mutant co-inoculation, and 8089 for the KO16 mutant co-inoculation. Among these, 164 core bacterial ASVs were shared across all four treatment groups (including the single-inoculation groups mentioned previously) (Figure 4a). Analysis of the core community in the wild-type T30 and *F. verticillioides* co-inoculation group revealed *Roseococcus* sp. and *Camelimonas* sp. as the dominant taxa. In contrast, the core community in the KO16 and *F. verticillioides* co-inoculation group was dominated by *Pelomonas* sp. (Figure 4b).

### 3.4. Effect of the Hyd1-Overexpressing T. harzianum Strain on Potential Metabolic Pathways in the Root Microbiome

**Figure 5:**
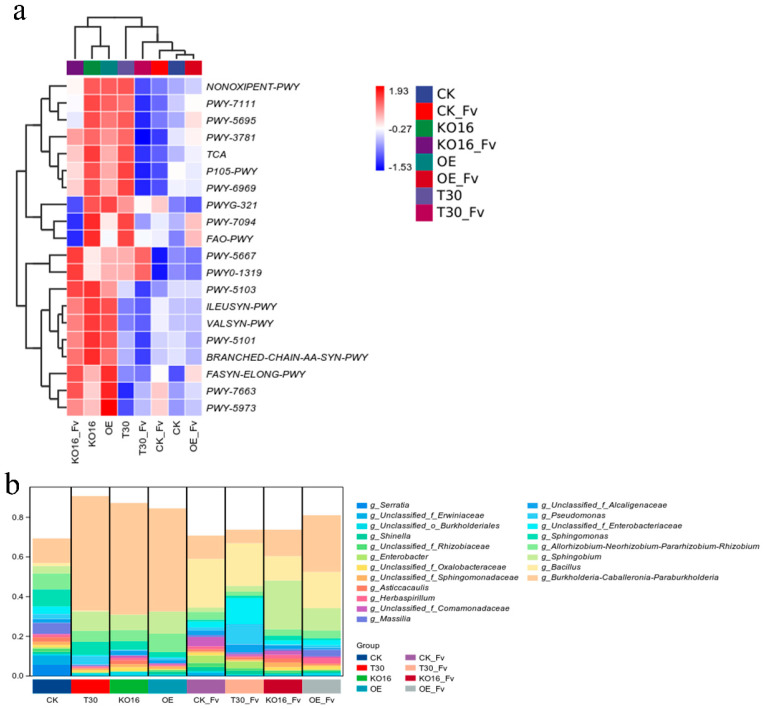
Metabolism and functional characteristics of the root bacterial community under inoculation with *hyd1* strains and *F. verticillioides* treatments. (**a**) Heat map analysis showing the bacterial genus metabolic pathways in maize roots. Red colors indicate higher relative abundance; blue colors indicate lower relative abundance. NONOXIPENT-PWY: Pentose phosphate pathway. PWY-7111: Pyruvate fermentation to isobutanol. PWY-5695: Purine-nucleoside phosphorylase. PWY-3781: Aerobic respiration I. TCA: TCA cycle I. P105-PWY: TCA cycle IV. PWY-6969: TCA cycle V. PWYG-321: Mycolate biosynthesis. PWY-7094: Fatty acid salvage. FAO-PWY: Fatty acid beta-oxidation I. PWY-5667: CDP-diacylglycerol biosynthesis I. PWY0-1319: CDP-diacylglycerol biosynthesis II. PWY-5103: L-isoleucine biosynthesis III. ILEUSYN-PWY: L-isoleucine biosynthesis I (from threonine). VALSYN-PWY: L-valine biosynthesis. PWY-5101: L-isoleucine biosynthesis II. BRANCHED-CHAIN-AA-SYN-PWY: Superpathway of branched-chain amino acid biosynthesis. FASYN-ELONG-PWY: Superpathway of fatty acid biosynthesis initiation. PWY-7663: Gondoate biosynthesis (anaerobic). PWY-5973: Cis-vaccenate biosynthesis. (**b**) Revealing the bacterial community involved in metabolic pathway patterns.

The potential functional roles and metabolic pathways of the root endophytic bacterial communities were predicted using PICRUSt2 based on the MetaCyc database. The functional profiles of the root microbiota differed across treatments, allowing us to cluster the plant treatments and identify the predominant functional categories (Figure 5a). These categories comprised seven main types—biosynthesis, degradation, detoxification, generation of precursor metabolite and energy, glycan pathways, macromolecule modification, and metabolic clusters—represented by 48 specific functional pathways (Figure 5a).

Among these, the FASYN-ELONG-PWY pathway (fatty acid biosynthesis) was possibly identified as the bacterial metabolic pathway in both the *Trichoderma* OE-*hyd1* treatment and the treatment co-inoculated with *F. verticillioides* (Figure 5a). Furthermore, in the roots co-inoculated with both OE-*hyd1* and *F. verticillioides*, the genera *Burkholderia*-*Caballeronia*-*Paraburkholderia*, *Bacillus*, and *Sphingobium* are hypothesized to be the primary contributors to the regulation of this fatty acid biosynthesis pathway (Figure 5b).

### 3.5. Endophytic Bacillus Strains Inhibit Growth of F. verticillioides and Promote Maize Growth

**Figure 6:**
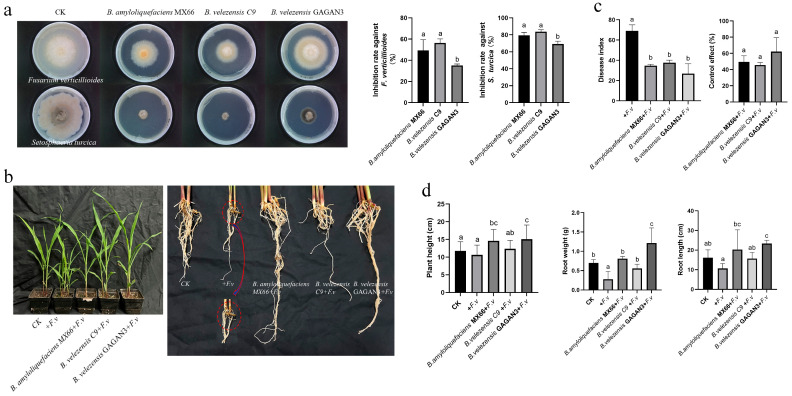
Suppressive activities of root-isolated bacteria against the root rot disease pathogen *F. verticillioides*. (**a**) The inhibitory effect of 3 *Bacillus* strains on *F. verticillioides* and *E. turcicum* mycelial growth in culture assays. (**b**) Overview of the growth status and root phenotypes of maize seedlings (Zhengdan958, cultivar) at 4 weeks after treatment with sterile water as a control (CK), *F. verticillioides* (*F.v*), *F.v* with *B. amyloliquefaciens* MX66, *B. velezensis* C9, or *B. velezensis* GAGAN3 (Red arrow indicates the rotten maize roots infected with *F. verticillioides*). (**c**) Disease index and control effect on maize seedling root rot for treatment with *F. verticillioides* or combined with 3 *Bacillus* isolates (3 *Bacillus* isolates + *F. verticillioides*). (**d**) The plant height, root length, and root fresh weight of maize per plant in different treatments. Values are means ± SDs (*n* = 3). Different letters indicate significant differences (*p* < 0.05) based on Tukey’s least significant difference test.

To investigate the role of *Bacillus amyloliquefaciens* MX66, *Bacillus velezensis* C9, and *Bacillus velezensis* GAGAN3 in suppressing root diseases, we conducted both in vitro antagonistic assays and pot experiments. Sterile culture filtrates of *B. velezensis* and *B. amyloliquefaciens* were prepared to evaluate their efficacy as biocontrol agents. As shown in Figure 6a, the inhibition rates against *F. verticilliodes* ranged between 35% and 56.3%, while those against *Exserohilum turcicum* were 79.4%, 83.7%, and 69.2%, respectively. The results demonstrated that all three *Bacillus* strains exhibited antagonistic activity against *F. verticillioides*.

Further analysis of the pot experiments revealed that treatment with *B. amyloliquefaciens* MX66, *B. velezensis* C9, and *B. velezensis* GAGAN3 significantly promoted maize growth. Specifically, these treatments increased plant height by 37.4%, 16.2%, and 41.6%, and root length by 89.8%, 46.9%, and 118.5%, respectively, compared to the pathogen-only control. Additionally, they enhanced root weight by 1.91-, 1-, and 3.37-fold, respectively (*p* < 0.05; Figure 6d). The same bacterial inoculations also effectively suppressed root disease. The disease index of *F. verticillioides* root rot was reduced by 46.6% to 55.5% (Figure 6c), and correspondingly, the pathogen levels in the roots were significantly lowered (Figure 6c). These results demonstrated that supplementing with *B. amyloliquefaciens* MX66, *B. velezensis* C9, and *B. velezensis* GAGAN3 enhanced the root’s ability to suppress *F. verticillioides* infection.

### 3.6. Defense Signaling Pathways of Maize Activated by Endophytic Bacteria Against Fusarium Root Rot

**Figure 7:**
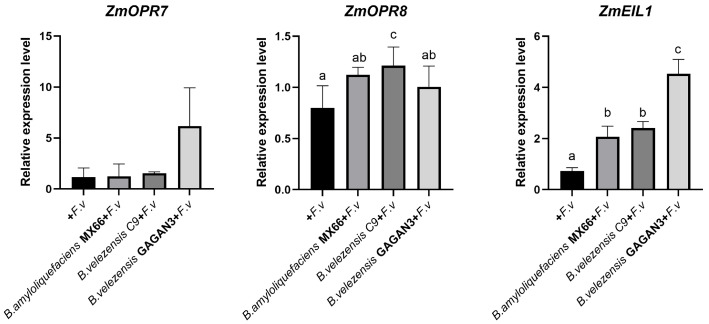
Effects of beneficial endophytic *Bacillus* isolate inoculations and *F. verticillioides* (*F.v*) infection on the expression of defense genes in maize root. Relative expression levels of maize *ZmOPR7, ZmOPR8*, and *ZmEIL1*, as determined by RT-qPCR. Values are means ± SDs (*n* = 3). Different lowercase letters above the columns indicate significant differences (*p* < 0.05) as calculated by one-way ANOVA and indicated by Tukey’s test.

To evaluate the impact of the isolated endophytes on the plant defense system, we inoculated maize with *B. amyloliquefaciens* MX66, *B. velezensis* C9, and *B. velezensis* GAGAN3 strains previously shown to antagonize *F. verticillioides* of root rot disease (Figure 7). In the presence of *F. verticillioides*, all three *Bacillus* treatments enhanced plant resistance to the pathogen and promoted plant growth compared to the non-inoculated control. Quantitative PCR analysis revealed that the expression of defense-related genes was significantly upregulated in the roots co-inoculated with *Bacillus* strains (MX66, C9, or GAGAN3) and *F. verticillioides*, compared to roots inoculated with *F. verticillioides* alone. Specifically, *ZmOPR7*, *ZmOPR8* and *ZmEIL1* were upregulated by 5.4-, 1.5-, and 4.6-fold, respectively (Figure 7). This indicated that the presence of *Bacillus* species primed a stronger JA/ET-mediated defense response in maize roots.

## 4. Discussion

Fungal diseases caused by *F. verticillioides* threaten crop production, human and environmental safety, and *Trichoderma* agent has been widely used worldwide to combat soilborne diseases in maize; however, the role of endophytic microbes in the root specifically induced by *Trichoderma* agent in the suppression of Fusarium root rot still remains unclear. The plant microbiome composition was found to be altered by pathogen infection, and beneficial microbes promoting disease resistance were recruited during growth [30]. It has been documented that *T. harzianum* T30 adheres to and colonizes maize roots through the *hyd1* protein, which can trigger a series of defense responses in maize and induce the maize to exhibit systemic resistance (ISR) to foliar disease [27]. Furthermore, research confirmed that *hyd1*, as an elicitor of induced systemic resistance (ISR) in maize, was localized on maize (*Zea mays*) root cell plasma membranes, interacting directly with root ubiquilin 1-like [31]. However, little is known about the way in which the *Trichoderma* elicitor *hyd1* induces changes in endophytic microorganisms related to Fusarium root rot. The seed application of *Trichoderma gamsii* induced the seedlings’ resistance against *F. verticillioides* by upregulating the transcript levels of the marker genes *ZmLOX10*, *ZmAOS*, and *ZmHPL*, and promoted maize seedling growth, with a reduction in fumonisin concentration [32]. The colonization of *Trichoderma virens* in maize plants triggered the resistance-related JA pathway, inhibiting infection by the pathogen *Colletotrichum graminicola* [33]. *Trichoderma asperellum* AC.3 inoculation inhibited *Peronosclerospora* spp. and enhanced maize growth by activating a defense-related biochemical response [24]. Similar effects have also been observed in other *Trichoderma* elicitors; for example, the deletion of *epl1* and *sm1* in *T. atroviride* and *T. virens* induced lower plant resistance against the pathogen, respectively. The overexpression of *epl1* and *sm1* in *Trichoderma* strains increased the resistance of the plants to pathogens. The expression of peroxidase- and α-dioxygenase-encoding genes was upregulated by *Epl1* and *Sm1* of *Trichoderma* spp, protecting against a tomato pathogen [34]. Consistent with previous findings, expression of the *EPL1* elicitor gene in *T. atroviride* triggered plant resistance against phytopathogens in *Arabidopsis thaliana* plants by induced increasing expression of SA- and JA-related genes [35].

Our study revealed that colonization by the *hyd1*-overexpressing strain of *Trichoderma* could promote maize’s growth and inhibit the infection of its seedling roots by the target pathogen *F. verticillioides*. Meanwhile, the endophytic bacterial community also changed compared with that observed with the treatment inoculated with the *Trichoderma hyd1*-deletion mutant during maize’s growth stages. *Pseudomonas* spp. and *Bacillus* spp. are among the most predominant genera of plant-beneficial bacteria specifically induced by the *hyd1* elicitor of *Trichoderma*. The endophytic bacterial genera *Sphingomonas* and *Pseudomonas* are useful for plants. In this study, functional prediction showed that the bacterial biosynthesis metabolic pathway was related to OE-*hyd1* treatments, suggesting the potential ability for secondary metabolism in the root endophytic bacteria in response to Fusarium disease.

Previous studies have shown that the bacterial microbiome plays a more crucial role in influencing the severity of soilborne diseases [17,36,37]. This study demonstrated that the inoculation of *hyd1*-overexpressing *T. harzianum* alone and combined with *F. verticillioides* may stimulate the proliferation of *Pseudomonas, Sphingomonas, Flavobacterium*, and *Streptomyces*, playing a potential role in maize growth promotion and antagonistic activity against pathogenic fungi, with biocontrol agents possibly contributing to the growth of beneficial bacteria through symbiotic interactions and metabolite sharing. A pot experiment further revealed that *Bacillus* was able to significantly suppress maize root rot and promote root growth. This study found that microbial communities were correlated with the control of root rot. The α diversity index of root endophytic bacteria exhibited an insignificant change between the treatments of co-inoculation with *F. verticillioides* and *hyd1*-overexpressing *T. harzianum* and the *hyd1*-deletion mutant KO16, which is different from other findings [38]. The colonization of *Trichoderma* strains with *hyd1* (wild-type and OE-*hyd1*) in maize roots showed a comparable effect on bacterial community composition, higher than KO16 treatment. It was supposed that the *hyd1* elicitor in the *T. harzianum* strain may modulate the plant–microbe interaction in such a way that this signaling favors the recruitment of beneficial endophytic microorganisms, which in turn help the plant to combat root rot.

The results suggest that *Trichoderma hyd1* and *F. verticillioides* infection can be the driving modulators of root endophytic bacterial community composition and diversity. Specifically, *Bacillus* genus abundance was enhanced in the root endophytic bacterial community under OE-*hyd1* with infection with *F. verticillioides*; this may represent the active recruitment of beneficial microbes by the host in response to disease stress. *Bacillus* species are widely studied for their antagonistic potential [39,40,41,42]. Several strains of *Bacillus* have been evaluated for their efficacy in controlling soilborne diseases [25,43]. For example, *B. velezensis* LDO2 inhibited the growth of peanut fungal and bacterial pathogens and promoted peanut root growth [44]. *B.subtilis* inoculation enriched distinct bacterial taxa and controlled *Fusarium verticillioides*-caused disease [45]. Another study observed that the plant-associated bacterium *Bacillus altitudinis* can confer resistance to soybean seed rot [31]. Plants have revealed the promising effects of decreasing rhizome rot disease caused by *Pectobacterium carotovorum* inoculated with *B. subtilis* [46]. Additionally, endophyte *B. subtilis* treatments were able to restrict the invasion of *Magnaporthiopsis maydis* in host maize roots [47]. The research elucidated that three endophytic bacteria, *B. amyloliquefaciens* MX66, *B. velezensis* C9, and *B. velezensis* GAGAN3, isolated from the maize roots, were able to inhibit infection by *F. verticillioides* and promote plant root growth. The antifungal substances probably include organic acids, lytic enzymes, hydrogen peroxide, diacetyl, and peptides produced by *Bacillus*. Previous work revealed that the introduction of beneficial endophytic bacteria resulted in positive plant-microbiome feedback, that is, via lowering the pathogen pressure by enriching the environment with beneficial antagonistic taxa while decreasing the pathogen density, suppressing plant disease [48,49].

There have been recent advances in the beneficial microbial colonization of roots to enhance the host’s defense against pathogens. These include inducing the JA and ethylene-ISR response to achieve metabolic defense in a manner dependent on host branched-chain amino acids and generating enzymes to scavenge reactive oxygen species [50,51,52]. Meanwhile, nutrient uptake and plant root structure transformation were found to be involved in the mechanism [53]. Previous studies have indicated that beneficial bacteria, including *Stenotrophomonas*, *Pseudomonas*, and *Streptomyces*, significantly activate JA- and SA-related pathways and improve the disease resistance of host plants [37,54]. Endophytic bacterial *Pseudomonas rhizophila* CTR8 from cotton roots upregulates JA-biosynthesis defense genes expression and promotes cotton seedling growth and resistance to *Verticillium* wilt [55]. Another study has demonstrated that the pretreatment of roots with beneficial bacterial isolates of *B. velezensis* FZB42 restricts *Verticillium longisporum* Vl43 infection in roots and systemically induces the JA and ET hormone signal transduction pathways in rapeseed [56]. JA-dependent ISR pathways are important components of plant defense transduction networks against pathogen attack. In addition, it has been reported that plant-associated microorganisms can prevent pathogen infections through the activated immunity of plants [57]. It is well known that root endophytic microorganisms can produce a variety of signaling molecules, such as lipopolysaccharides, peptidoglycans, and chitin fragments. These molecules are recognized by pattern recognition receptors (PRRs) on the surface of maize root cells, thereby activating the plant’s basic immune response, namely pathogen-associated molecular pattern-triggered immunity (PTI). However, microbial analysis has been constrained by partial *16S* rRNA sequencing, which has restricted the identification of fungi and viruses and the annotation of bacterial taxa at the species level, resulting in uncomprehensive acknowledge about microbial community composition, structure, and diversity. It should be noted that functional predictions made using partial 16S regions through PICRUSt _MetaCyc pose the problem that characterized metabolic traits related to species- and genus-level taxonomic classification may not exactly correspond to changes in microbial functional genes, which gives rise to ambiguity in microbial function metabolic pathway prediction. Further, the incorporation of complementary techniques, including metagenomics, instrumentally facilitates this study. The microbial molecular and metabolic changes and resistance pathway mechanisms (with respect to *Fusarium* disease) induced by *Trichoderma hyd1* and the interaction between *Trichoderma hyd1* and beneficial microorganisms colonizing maize roots are poorly understood and require further experimental investigation. The balanced relationship between plants and the root microbial community is considered crucial for promoting plant growth and suppressing plant diseases. In addition, it is essential for understanding the effects of the elicitor *hyd1* from *T. harzianum* on the reshaped endophytic microbial community related to the immunity response of maize roots. Endophytic *B. amyloliquefaciens* MX66, *B. velezensis* C9, and *B. velezensis* GAGAN3 are regarded as major members of the root bacterial microbial community. These findings are consistent with the previous study that reported that synthetic communities suppress *Astragalus* root rot by activating JA- and ISR-related enzyme activities [58].

## 5. Conclusions

To our knowledge, root colonization by the *T. harzianum* strain overexpressing the elicitor *hyd1* (OE-*hyd1*) reshapes the maize root endophytic microbial community, promoting root growth and suppressing disease under greenhouse conditions, highlighting the role of the *hyd1* elicitor during the plant–*Trichoderma* and endophytic bacterial interaction. *B. amyloliquefaciens* MX66, *B. velezensis* C9, and *B. velezensis* GAGAN3 were identified as major members of endophytic bacteria induced by the elicitor *hyd1* from *T. harzianum*, which were responsible for enhancing the host immunity response against Fusarium root rot. This study provides insights into the multiple beneficial effects of the elicitor *hyd1* from *T. harzianum* in the recruitment of endophytic beneficial microbes in roots and then their integration to enhance maize growth and resistance against *F. verticillioides* root rot in maize.

## Data Availability

The original contributions presented in this study are included in the article. Further inquiries can be directed to the corresponding author(s). *16S* rRNA sequencing data can be accessed on SRA under the accession PRJNA1457710. Please visit this link to access the *16S* rRNA sequencing data, https://dataview.ncbi.nlm.nih.gov/object/PRJNA1457710?reviewer=sjssepl506h72etbfb68ia61hl (accessed on 26 April 2026).

## Supplementary Materials

The following supporting information can be downloaded at: https://www.mdpi.com/article/10.3390/jof12060395/s1, Figure S1: The graph scheme of the experimental treatment and methods in the study; Table S1: Primers used in the experiment; Table S2: The sequencing metrics in maize root samples.
