# Supplementary material for: The Role of Trichoderma harzianum Elicitor Hyd1 in Inducing the Maize Endophytic Microbial Community and Bacillus Strains Against Maize Root Rot
Source: J Fungi (Basel). 2026 May 30;12(6):395. doi: 10.3390/jof12060395 (PMC13301929; doi:10.3390/jof12060395)
Supplement: Supplementary file 1 [file jof-12-00395-s001.zip › Supplementary.pdf]

Journal of *Fungi*

MDPI

Supplementary Information

Article title: The Role of *Trichoderma harzianum* Elicitor *Hyd1* in Inducing Maize Endophytic Microbial Community and *Bacillus* Strains Against Maize Root Rot

The following Supplementary information is available for this article:

Figure S1. The graph scheme of the experimental treatment and methods in the study.

Table S1. Primers used in the experiment.

Figure S1. The graph scheme of the experimental treatment and methods in the study.

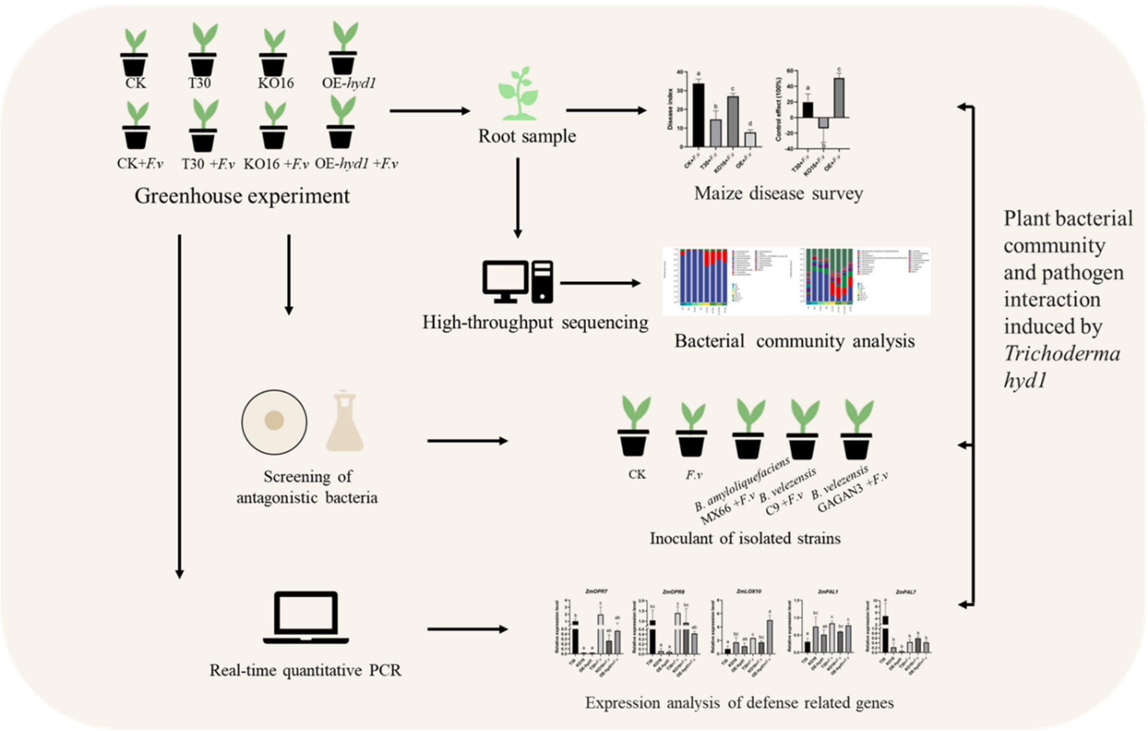

Table S1. Primers used in the experiment.

**Primer name**	**Sequence (5′-3′)**	**Remark**
ZmUBI-F	GGAAAAACCATAACCCTGGA	qPCR
ZmUBI-R	ATATGGAGAGAGGGCACCAG	qPCR

ZmPAL1-F	AGAACGCCAAGGAGAAGAGG	qPCR
ZmPAL1-R	GAAGAAAGAGCAACGCCACA	qPCR
ZmPAL7-F	AGAGAAAATACAAGGAGCAGAAGAC	qPCR
ZmPAL7-R	GATTCGCATACTCGAAAACCTA	qPCR
ZmLOX10-F	TCTGTCTGAGCTGAGGACGTA	qPCR
ZmLOX10-R	CACAAAGTAACTTCATTATTGAGGA	qPCR
ZmOPR7-F	TCTCTTCTCGCCGTACCAGA	qPCR
ZmOPR7-R	AACAATCGCGGCATTACCCA	qPCR
ZmOPR8-F	CCATAAACGCCATCAAAGCA	qPCR
ZmOPR8-R	GAGCCACCGTACTCGTCAGC	qPCR
ZmEIL1-F	AGCCAATCAGGCAGTGAACAAT	qPCR
ZmEIL1-R	CCTCTCCATGATGGTGACGCTG	qPCR
